# Perceived Spatial Environment and Outdoor Activity in Middle-Aged and Older Chinese Adults: A Cross-Sectional Examination of Affective and Cognitive Mediating Pathways

**DOI:** 10.3390/bs16071139

**Published:** 2026-07-07

**Authors:** Bojing Liao, Bo Li, Xinxin Lin, Qiantong Ouyang

**Affiliations:** 1 Institute of Creativity and Innovation, Xiamen University, Xiamen 363105, China; lbj@xmu.edu.cn (B.L.); 36320232200032@stu.xmu.edu.cn (X.L.); 36320242200174@stu.xmu.edu.cn (Q.O.); 2 School of Design & Innovation, Xiamen University Tan Kah Kee College, Zhangzhou 363123, China

**Keywords:** perceived spatial environment, place attachment, outdoor activity, middle-aged and older adults, parallel mediation, age-friendly community

## Abstract

**Background:** Outdoor activity is essential for healthy aging, yet the affective, cognitive, and behavioral pathways linking perceived neighborhood environment to outdoor activity in middle-aged and older Chinese adults remain underexplored. **Objective:** This cross-sectional study examined associations between perceived spatial environment and outdoor activity, and tested five candidate mediators in a parallel-mediation framework, among community-dwelling adults aged ≥ 50 in Xiamen, China. **Methods:** Of 251 returned questionnaires, 99 cases with inconsistent response patterns and 12 high-leverage cases (Cook’s *D* > 3/*n*) were excluded, yielding *N* = 140. Hierarchical regression and non-parametric bootstrap mediation (5000 resamples; bias-corrected 95% Cis) were conducted, with sensitivity analyses, Fornell–Larcker discriminant-validity assessment, and Harman’s single-factor test for common-method bias. **Results:** Perceived spatial environment was strongly associated with outdoor activity (β = 0.96, *p* < 0.001). Bootstrap analyses identified four significant indirect pathways—place attachment (0.26 [0.13, 0.39]), perceived social environment (0.21 [0.11, 0.31]), service environment (0.18 [0.09, 0.28]), and attitudinal preferences (0.17 [0.05, 0.29])—whereas the a priori hypothesized behavioral mediator, social interaction, did not reach statistical significance (0.10 [−0.000, 0.205]). Sensitivity analyses confirmed coefficient stability across outlier-trimmed and untrimmed samples. **Conclusions:** Findings are consistent with an environment–behavior model in which affective and cognitive constructs—particularly place attachment—appear to be more proximal mediators than enacted social-interaction frequency. Given high inter-construct correlations (limited discriminant validity) and a self-selected community-active sample, results are best interpreted as hypothesis-generating and require replication with longitudinal and multi-method designs.

## 1. Introduction

The global demographic transition toward an aging population is fundamentally restructuring the behavioral demands placed upon urban ecosystems. The World Health Organization (WHO) projects that the global cohort aged 60 years and above will reach 1.4 billion by 2030 (World Health Organization, 2020). China operates at the vanguard of this transition: by the end of 2023, 296.97 million residents—accounting for 21.1% of the national populace—were aged 60 or older (National Bureau of Statistics of China, 2024). As “aging in place” solidifies as a central policy paradigm, sustaining everyday outdoor activity has crystallized as a primary behavioral marker for healthy aging.

Outdoor activity—encompassing out-of-home locomotion and recreational engagement—transcends mere physical movement. It is inextricably linked to preserved physical functioning and self-rated health (Kerr et al., 2012), sustained mobility, and functional independence (Yen et al., 2014). Furthermore, contemporary systematic reviews substantiate its profound reliance on the configuration of the built environment (Barnett et al., 2017). Consequently, outdoor activity offers a highly tractable behavioral outcome through which the complex dynamics of person–environment fit can be empirically validated.

Historically, environment–behavior research has anchored its methodologies in objective, GIS-derived spatial metrics. The foundational “5D” framework (density, diversity, design, destination accessibility, distance to transit) remains a staple for modeling travel trajectories and walkability (Cervero & Kockelman, 1997). However, for older adults experiencing normative sensory and physical declines, these macro-level spatial indicators often lack the requisite granularity to capture the micro-scale features that dictate day-to-day environmental usability. Perceived walkability, subjective safety, and the accessibility of supportive infrastructure critically govern mobility behaviors in later life (Rosso et al., 2011). Recent studies affirm robust associations between the perceived physical attributes of neighborhoods and leisure-time physical activity of older adults (Van Cauwenberg et al., 2018). Particularly within high-density Asian metropolises, the perceived spatial environment emerges as an exceptionally potent correlate of active living (Cerin et al., 2023). To that end, the perceived spatial environment often exhibits greater behavioral salience for older populations than purely objective geospatial indices alone.

While literature linking neighborhood environments to physical activity has matured, three critical deficits frame this inquiry. First, empirical evidence from dense East Asian contexts—where neighborhood morphology, intergenerational living arrangements, and micro-public spaces diverge fundamentally from Western suburban paradigms—remains notably sparse (Cerin et al., 2023). Second, parallel-mediation models simultaneously evaluating affective (place attachment), cognitive (attitudinal preferences), behavioral (social interaction), and contextual (perceived social and service environments) pathways are exceptionally rare; conventional scholarship overwhelmingly isolates these mechanisms. Third, existing research disproportionately targets adults aged ≥ 65, systematically neglecting the pre-onset window (50–59 years)—the critical phase of maximum behavioral plasticity for active-aging interventions (Bauman et al., 2016).

Although conventional aging research disproportionately anchors on adults aged ≥ 65, both the WHO Active Ageing framework (World Health Organization, 2002, 2020) and China’s 14th Five-Year Plan for Healthy Aging (2022–2025) explicitly target adults aged ≥ 50. Three imperatives justify this expanded demographic threshold. First, precipitous declines in physical activity, social-network breadth, and spatial mobility typically manifest in the late 50s rather than at 65 (Bauman et al., 2016). Second, preventative interventions achieve maximum efficacy prior to multi-morbidity onset, establishing the 50–59 cohort as an indispensable pre-geriatric window. Third, incorporating this demographic captures the unbroken continuum of mid-to-late-life behavioral transitions within a unified analytical framework. Consequently, we establish age ≥ 50 as the baseline inclusion criterion, explicitly operationalizing this population as “middle-aged and older adults” throughout.

To delineate why environmental perception precipitates actual outdoor behavior, the ecological theory of aging provides a foundational heuristic: late-life behavior manifests through the dynamic interface between environmental press and individual competence (Lawton & Nahemow, 1973). Complementing this from an urban-design perspective, Gehl posits that the spatial quality of public realms directly modulates the volume and duration of necessary, optional, and social activities nested within them (Gehl, 2010).

Above mentioned complementary frameworks conceptualize community spaces not as passive backdrops, but as active behavioral settings that afford repeated encounters, informal communication, and localized support networks. The social-capital theory extends this logic, emphasizing that such recurring, localized interactions form the bedrock of collective trust and reciprocity (Putnam, 2000). Empirically, heightened social cohesion and community belonging reliably predict well-being among older community-dwelling residents (Cramm & Nieboer, 2015).

Despite this theoretical convergence, the mediating behavioral pathway—whereby the perceived spatial environment catalyzes social interaction to ultimately foster outdoor activity—remains critically underexplored within the Chinese urban context. This omission is substantive. High-density Chinese metropolises are characterized by constrained private living spaces and the intensive, shared utilization of community-level outdoor amenities. Under these architectural conditions, courtyards, building thresholds, and micro-plazas operate as vital behavioral nodes where walking, resting, exercising, and social exchange are tightly integrated.

To bridge this empirical gap, the present study utilizes cross-sectional survey data from 140 community-dwelling residents aged 50 years and older in Xiamen, China. The overarching research question asks: Within high-density Chinese communities, is the perceived spatial environment associated with older adults’ outdoor activity directly, indirectly via neighborhood social interaction, or both?

We formally test four hypotheses:

**H1.**  *Perceived spatial environment is positively associated with outdoor activity.*

**H2.**  *Perceived spatial environment is positively associated with neighborhood social interaction.*

**H3.**  *Neighborhood social interaction mediates the relationship between perceived spatial environment and outdoor activity.*

**H4.**  *Place attachment, attitudinal preferences, perceived social environment, and perceived service environment serve as parallel mediators of the same association.*

This study advances behavioral science research across three dimensions. First, it provides a rigorous empirical test of the perception–interaction–activity pathway within high-density Chinese communities—an architectural context historically underrepresented in spatial-behavioral analyses. Second, it explores complementary mechanisms (e.g., place attachment, spatial attitudes and preferences, and service environments) to determine whether additional psychological or contextual pathways operate in tandem with the focal social pathway. Third, it translates behavioral evidence into actionable interventions for age-friendly community renewal.

The remainder of the article is structured as follows. Section 2 delineates the study area, sampling protocol, measurement instruments, and analytical strategy. Section 3 details the descriptive statistics, reliability and validity assessments, correlation analyses, as well as the hierarchical regression and mediation modeling results. Section 4 and Section 5 discusses theoretical ramifications, practical applications and limitations. Section 6 provides concluding remarks.

## 2. Materials and Methods

### 2.1. Study Area and Sampled Communities

Xiamen, a sub-provincial coastal municipality in Fujian Province, China, serves as the empirical setting for this study. As of 2024, the city reported a resident population of approximately 5.35 million, with 643,000 individuals (12.02%) aged 60 years or older (Xiamen Municipal Civil Affairs Bureau & Xiamen Office for Aging, 2025). The selection of Xiamen was predicated on three primary considerations.

First, its subtropical marine climate facilitates year-round outdoor engagement, establishing a behaviorally rich context for scrutinizing environment–activity dynamics. Second, the urban fabric encompasses highly heterogeneous residential typologies—ranging from pre-2000 open blocks and traditional work-unit compounds to post-2000 commercial gated communities—thereby ensuring robust variance in environmental exposures within a unified municipal context. Third, Xiamen’s proactive implementation of age-friendly community initiatives positions it as an exemplary case study capable of yielding actionable, policy-relevant behavioral insights.

**Figure 1:**
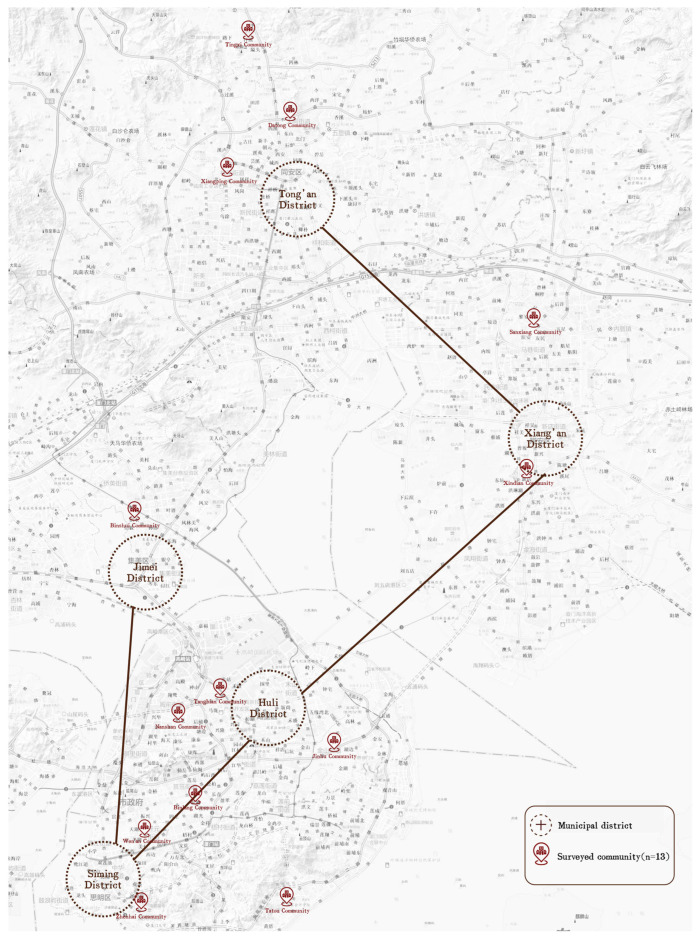
Geographic distribution of the 13 sampled community clusters in Xiamen (locations are approximate).

A purposive sampling strategy was deployed across four districts (Siming, Huli, Jimei, and Tongan) to select 13 communities, capturing critical variations in housing-stock vintage (pre-2000 versus post-2000), governance structures (work-unit versus commercial gated), and the presence of prior age-friendly retrofitting. For instance, Zhenhai Community represents a pre-2000 work-unit compound characterized by a high floor-area ratio, constrained green space, and an absence of recent spatial interventions; conversely, Xiangping Community epitomizes a post-2000 commercial gated development equipped with well-maintained community centers and age-friendly amenities. The approximate spatial distribution of these communities is illustrated in Figure 1.

### 2.2. Sampling and Data Collection

The target demographic comprised permanent residents aged 50 years and older who had resided in the sampled communities for a minimum of six months. The deliberate inclusion of the 50–59 age bracket captures a pre-geriatric (early-aging) cohort whose spatial routines and environmental dependencies are already being actively shaped by aging-in-place trajectories. Incorporating this demographic continuum allows the behavioral shifts spanning middle to later adulthood to be evaluated within a unified analytical paradigm.

A community-assisted convenience sampling protocol was executed. Participants were intercepted at key behavioral nodes, including community activity centers, local parks, chess rooms, and building thresholds. Inclusion criteria necessitated sufficient cognitive capacity to complete the survey independently or via interviewer administration; individuals with severe auditory, visual, or documented cognitive impairments were excluded.

Data collection proceeded between September 2025 and March 2026. A team of five rigorously trained graduate research assistants conducted face-to-face questionnaire administrations. Informed consent was systematically obtained prior to survey initiation. To accommodate respondents with limited literacy, assistants verbally administered the items. All completed instruments underwent immediate on-site verification, and participants were compensated with a nominal gift. The average administration duration was 20–25 min.

Of 280 distributed questionnaires, 251 were returned (an 89.6% response rate) and subjected to two sequential screening steps. **Step 1 (response-pattern screening) separated valid returns**—Pattern 1 (n=152), which retained complete data across all core constructs and cleared three attention checks (response-time thresholds, monotonic-pattern detection, and reverse-coded item consistency)—from incomplete or failed returns (Pattern 2, n=99; excluded). **Step 2 (multivariate-outlier screening) evaluated Cook’s distance (**D**) for the primary regression model.** Applying Stevens’ (2009) conservative threshold of D>3/n (=3/152≈0.0197), we excluded 12 high-leverage observations, yielding a final analytic sample of N=140.

Table A1 details the attrition analysis comparing the analytic sample (N=140) against all excluded respondents (N=111). Compared to the excluded cohort, the final sample skewed significantly toward older (Δage=+0.33, t=3.30, p=0.001), less-educated (Δedu=−0.98, t=−5.95, p<0.001), less-healthy (Δhealth=−0.64, t=−5.08, p<0.001), and lower-income (Δincome=−0.28, t=−2.47, p=0.014) individuals. Conversely, gender ($\chi^{2}=3.11$, p=0.078) and chronic-disease status ($\chi^{2}=1.22$, p=0.270) exhibited no statistically significant divergence. These systematic compositional variations must carefully frame the subsequent interpretation of external validity. Pattern 1 was retained as the primary analytical dataset.

To optimize the robustness of the regression estimates and mitigate the influence of multivariate outliers, Cook’s distance ($D_{i}$) was calculated for the primary regression model predicting outdoor activity. Adhering to Stevens’ (2009) conservative criterion, a strict exclusion threshold was implemented:

(1) $$D_{i}>\frac{3}{n}$$

For the 152 retained cases, this equated to a threshold of 3/152≈0.0197. Twelve cases exceeding this parameter were excluded, yielding a final, highly curated analytical sample of 140 valid cases.

### 2.3. Variable Measurement

All composite scales were adapted from validated instruments and back-translated into Mandarin Chinese following Brislin’s (1970) protocol. Source scales: Perceived Spatial Environment was adapted from the Neighborhood Environment Walkability Scale (NEWS; Cerin et al., 2006) and Lawton’s Multilevel Assessment Instrument (Lawton et al., 1982); Place Attachment from Williams and Vaske (2003); Neighborhood Social Interaction from Sampson et al. (1997); Outdoor Activity from the CHAMPS Activities Questionnaire (Stewart et al., 2001); Perceived Social Environment from the Neighborhood Cohesion subscale (Sampson et al., 1997); Service Environment from the WHO Age-Friendly Cities checklist (World Health Organization, 2007); Attitudinal Preferences was newly developed for this study based on focus-group input (*n* = 8 community elders).

The measurement model incorporated seven composite core constructs and five demographic covariates. Each composite variable was derived as the arithmetic mean of its constituent item scores, with higher values denoting a greater magnitude of the target construct.

**Perceived Spatial Environment (SPACE_ENV)** was operationalized using 26 items (P1_01–P1_26) evaluated on a 5-point Likert scale (1 = strongly disagree to 5 = strongly agree). This scale captures older adults’ subjective appraisals of community spatial quality across dimensions of accessibility, safety, ambient comfort, green infrastructure, resting amenities, and overall environmental affordances for outdoor activity.

**Neighborhood Social Interaction (SOCIAL_INT)** was quantified using a five-item inventory (M1–M5) scaled on a 1–4 frequency metric, where higher scores indicate a higher density of localized social exchanges.

**Outdoor Activity (OUTDOOR_ACT)** was assessed via six items (O1–O6) on a parallel 1–4 frequency scale, capturing the intensity and regularity of out-of-home engagement.

To interrogate supplementary mechanistic pathways (Appendix A), four additional latent constructs were evaluated. **Place Attachment (PLACE_ATTACH)** was captured via 12 items (5-point scale), while **Attitude and Preference (ATTITUDE)**, **Social Environment (SOC_ENV)**, and **Service Environment (SER_ENV)** were each measured using 8-item, 5-point scales. These variables were incorporated to ascertain whether complementary psychological, social, or service-oriented mechanisms operate in tandem with the primary social-interaction pathway.

The demographic covariates encompassed age group, gender, self-rated health, educational attainment, and chronic disease status. Age was partitioned into four strata (50–59, 60–69, 70–79, ≥80). Gender was coded dichotomously. Self-rated health and education were treated as ordinal variables, while the presence of chronic conditions was binary-coded.

### 2.4. Data Analysis

All statistical analyses were executed utilizing IBM SPSS Statistics (27.0.1). The analytical pipeline proceeded through five sequential phases.

First, descriptive statistics were generated to characterize the demographic profile of the sample and the distributional properties of the core constructs. Distributional normality was evaluated via skewness and kurtosis indices to confirm the appropriateness of subsequent parametric estimations.

Second, the psychometric integrity of the measurement scales was rigorously assessed. Internal consistency reliability was quantified using Cronbach’s α, while construct validity and the factorability of the item matrices were verified via the Kaiser–Meyer–Olkin (KMO) measure of sampling adequacy and Bartlett’s test of sphericity.

Third, bivariate relationships among the primary constructs were mapped using Pearson product-moment correlations. Demographic stratifications in the outcome variables were evaluated utilizing independent-samples *t*-tests (for dichotomous covariates: gender, chronic disease) and one-way analyses of variance (ANOVAs; for polytomous/ordinal covariates: age, education, self-rated health, income).

Fourth, hierarchical multiple regression modeling was deployed to sequentially evaluate the main effects (H1 and H2). Demographic covariates were introduced in Step 1 to adjust for baseline confounding, followed by the perceived spatial environment in Step 2, and neighborhood social interaction in Step 3. Robustness diagnostics, including Variance Inflation Factors (VIF) and Durbin–Watson statistics, were systematically scrutinized to rule out multicollinearity and ensure the independence of residuals.

Finally, the parallel-mediation framework (H3–H4) was evaluated via non-parametric bootstrapping (5000 resamples) with bias-corrected 95% confidence intervals (Cis), inferring statistical significance if the CI excluded zero (Hayes, 2018; Preacher & Hayes, 2008). Bootstrapping was favored over the Sobel test because it eschews normality assumptions for the indirect-effect sampling distribution, offers superior Type I error control in small-to-moderate samples, and represents the established discipline standard. All five candidate mediators—social interaction, place attachment, attitudinal preferences, and perceived social and service environments—were simultaneously estimated within a single multivariate model adjusting for age, gender, self-rated health, education, and chronic-disease status.

Construct validity was verified via Fornell–Larcker discriminant analysis, benchmarking inter-construct correlations against the $\sqrt{\mathrm{AVE}}$ (Fornell & Larcker, 1981), while common-method bias was evaluated via Harman’s single-factor test (Podsakoff et al., 2003). Additionally, sensitivity checks re-estimated all models on the unfiltered Pattern-1 sample (N=152) to confirm that substantive conclusions were not artifacts of Cook’s D outlier exclusions.

Age-stratified subgroup analyses were bypassed because cell sizes across the 50–59 (n=38), 60–69 (n=72), and 70–79 (n=22) strata fell below the minimum threshold required for medium-effect detection. Continuous age was consequently retained as a covariate throughout.

Statistical power was calculated using G*Power 3.1.9.7 (Faul et al., 2009) for multiple linear regression with six covariates, assuming α=0.05 and a target power of 0.80. The required sample size to detect a medium effect ($f^{2}=0.15$) was N=98, confirming that our final analytic sample (N=140) is adequately powered for both the primary regressions and the parallel-mediation framework. Conversely, detecting small effects ($f^{2}=0.05$) required N=273; our framework is consequently under-powered for minor effect sizes, a constraint explicitly acknowledged as a study limitation. Finally, because even the largest age-stratified subgroup (60–69 years, n=72) fell below the baseline N=98 threshold, stratified analyses were omitted from the revised manuscript to avoid under-powered statistical inferences.

**Figure 2:**
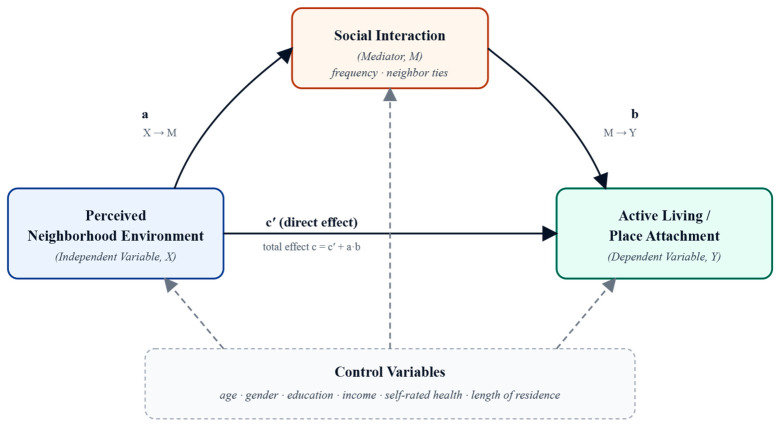
Conceptual mediation model linking perceived spatial environment (X), social interaction (M), and older adults’ active living/place attachment (Y). Path a represents the effect of the perceived environment on social interaction; path b represents the effect of social interaction on the outcome; path c’ denotes the direct effect after controlling for the mediator. The indirect (mediated) effect equals a × b, tested by the 5000-resample bootstrap mediation analysis.

The overarching theoretical framework guiding this mediation analysis is conceptualized in Figure 2.

## 3. Results

### 3.1. Participant Characteristics and Descriptive Statistics

Following stringent data screening and regression diagnostics, a highly curated cohort of 140 valid cases was retained for primary analysis. The cohort exhibited a balanced gender distribution (49.3% male, 50.7% female). Demographically, the sample was predominantly anchored by the 60–69 age bracket (51.4%), complemented by pre-geriatric (50–59 years; 27.1%), septuagenarian (70–79 years; 15.7%), and octogenarian or older (≥80 years; 5.7%) sub-cohorts. A vast majority of participants reported being married (91.4%).

Vocationally, retired personnel constituted the largest segment (47.9%). Socioeconomically, 44.3% reported a monthly pension or income below 3000 RMB, and approximately one-third (32.9%) possessed primary-level educational attainment or below. Regarding household composition, exactly half of the respondents co-resided exclusively with a spouse, whereas 25.7% resided in multi-generational households with children. Self-rated health profiles skewed moderate-to-favorable (40.0% reporting “good” health), coupled with a comparatively low prevalence of chronic medical conditions (19.3%). Comprehensive demographic characteristics are delineated in Table 1.

Distributional diagnostics confirmed robust normality across all core variables, with absolute skewness indices capped at 1.22 and kurtosis indices not exceeding 0.60 (Table 2). These parameters comfortably satisfy the distributional prerequisites for subsequent parametric estimations, encompassing Pearson product-moment correlations, *t*-tests, ANOVAs, and ordinary least squares (OLS) regression modeling.

### 3.2. Reliability and Validity Analysis

Preceding hypothesis testing, the psychometric integrity of the measurement instruments was systematically evaluated for reliability and construct validity (Table 3). Internal consistency proved excellent across all operationalized scales. The perceived spatial environment construct exhibited the highest reliability (α=0.966), followed successively by place attachment (α=0.931), outdoor activity (α=0.916), neighborhood social interaction (α=0.904), social environment (α=0.902), service environment (α=0.899), and attitude and preference (α=0.893).

Regarding factorability and construct validity, Kaiser–Meyer–Olkin (KMO) measures of sampling adequacy ranged from 0.888 to 0.970, and all Bartlett’s tests of sphericity were statistically significant (p<0.001). These diagnostics confirmed that the constituent items possessed substantial shared variance, robustly validating their suitability for subsequent inferential modeling.

An exploratory factor analysis via principal component analysis (PCA) with varimax rotation (KMO=0.913; Bartlett’s $\chi^{2}=8421.3$, p<0.001) extracted seven factors with eigenvalues > 1, cumulatively accounting for 71.4% of the total variance. All items loaded predominantly on their designated latent constructs (loadings: 0.62–0.89), establishing robust convergent validity as the Average Variance Extracted (AVE) ranged from 0.539 to 0.722, uniformly exceeding the 0.50 benchmark threshold (Fornell & Larcker, 1981).

### 3.3. Correlation Analysis

Bivariate associations among the seven latent constructs are delineated in Table 4. All pairwise product-moment correlations yielded positive coefficients of substantial magnitude, achieving statistical significance at the p<0.001 threshold. Crucially for the focal hypotheses, the perceived spatial environment exhibited exceptionally robust correlations with both outdoor activity (r=0.967) and neighborhood social interaction (r=0.932). Moreover, social interaction demonstrated a highly synchronous relationship with outdoor activity (r=0.919). Collectively, these bivariate metrics furnish strong preliminary substantiation for the hypothesized mechanistic pathway.

The extraordinarily high inter-construct correlations (r=0.877–0.967, all >0.85) substantially exceed typical behavioral research magnitudes, warranting stringent diagnostic scrutiny. Three distinct concerns require explicit acknowledgment. First, multiple coefficients surpass the 0.90 threshold conventionally flagging discriminant-validity violations (Kline, 2016), prompting the formal Fornell–Larcker verification (see Table A4). Second, the simultaneous, single-source cross-sectional design introduces potential common-method bias (CMB), evaluated via Harman’s single-factor test. Third, inherent semantic redundancies persist across focal constructs, notably the conceptual overlap between perceived spatial and service environments. Consequently, downstream regression and mediation estimates must be interpreted strictly as statistical associations subject to variance inflation, not definitive causal pathways. The elevated $R^{2}$ values thus likely conflate genuine empirical covariance with shared method variance and item-content collinearity.

### 3.4. Group Differences in Outdoor Activity and Social Interaction

Inferential diagnostics via independent-samples *t*-tests exhibited statistical invariance across genders; no significant differences were observed in outdoor activity, neighborhood social interaction, or the perceived spatial environment. Similarly, chronic disease status failed to induce systematic variance across any of the focal behavioral and perceptual outcomes.

Although descriptive group differences across age strata attained nominal statistical significance (outdoor activity: F=3.62, p=0.015; social interaction: F=3.82, p=0.011), these findings warrant no substantive interpretation. Given the highly asymmetrical cell sizes across the four age cohorts (n=38, 72, 22, 8), post hoc comparisons suffer from acute statistical power deficits, while Levene’s test confirmed significant variance heterogeneity in both ANOVA specifications (p<0.01). Consequently, age is retained strictly as a continuous control covariate in all downstream multivariate models, and we explicitly abstain from advancing any age-moderation claims. Other socioeconomic and health indicators—including educational attainment, self-rated health, and monthly income—yielded no statistically discernible group divergences. Notably, because Levene’s test underscored heteroscedasticity across several ANOVA formulations, these descriptive stratified insights are strictly framed as preliminary, exploratory evidence rather than the definitive baseline for hypothesis verification. To safeguard the validity of our core theoretical postulations against potential violations of homoscedasticity, the focal hypotheses were subsequently rigorously interrogated via multivariate regression frameworks that systematically adjusted for these demographic covariates. Detailed parameter estimates are compiled in Table 5.

### 3.5. Hierarchical Multiple Regression Analysis

A hierarchical multiple regression framework was deployed to systematically isolate the main effects of the perceived spatial environment and neighborhood social interaction on outdoor activity. To establish a foundational baseline, demographic covariates (age, gender, self-rated health, education, chronic disease) were introduced in Model 1. Model 2 incorporated the focal spatial predictor (perceived spatial environment), while Model 3 integrated the hypothesized social mediator (neighborhood social interaction). Finally, Model 4 constituted an exploratory expansion, assimilating the four supplementary psychological and contextual constructs (social environment, service environment, place attachment, attitude and preference).

As delineated in Table 6, the demographic block in Model 1 captured negligible variance in outdoor activity ($R^{2}=0.035,\;p=0.431$). Conversely, the inclusion of the perceived spatial environment in Model 2 yielded a profound enhancement in explanatory power ($R^{2}=0.940$, $\Delta R^{2}=0.904$, p<0.001). Within this stratum, spatial perception was strongly positively associated with outdoor engagement (β=0.960, p<0.001), thereby consistent with H1.

The subsequent integration of social interaction in Model 3 generated a modest yet statistically significant incremental gain in variance explained ($R^{2}=0.942$, $\Delta R^{2}=0.002$, p=0.020). Crucially, social interaction showed a significant positive association with outdoor activity (β=0.137, p<0.05). Concurrently, the main effect coefficient for the perceived spatial environment attenuated from β=0.962 to β=0.832 while retaining statistical significance. Although this step-wise attenuation is descriptively consistent with partial mediation, the formal parallel-mediation bootstrap (Section 3.6)—which adjusts simultaneously for the four affective and contextual mediators and for demographic covariates—did not support social interaction as a statistically significant mediator. The apparent mediation in this single-step model should therefore be interpreted with caution.

The exploratory assimilation of the four supplementary variables in Model 4 marginally elevated the total explanatory capacity to $R^{2}=0.953$. Within this expanded parameterization, place attachment exerted the most pronounced supplementary effect, whereas the social and service environments achieved only marginal significance. Given the conceptual proximity and high collinearity between these supplementary constructs and the primary focal variables, Model 4 is strictly interpreted as a heuristic exploration. Consequently, definitive hypothesis testing is anchored exclusively in Models 1–3, which map directly onto our theoretically postulated perception–interaction–activity pathway.

Finally, to ensure estimator stability, multicollinearity diagnostics were systematically evaluated across all steps. All Variance Inflation Factor (VIF) indices fell decisively below the conservative threshold of 10, confirming that multicollinearity did not compromise the structural integrity of the regression estimates.

A second hierarchical regression tested whether perceived spatial environment predicted neighborhood social interaction. With social interaction as the dependent variable, the demographic block produced negligible explanatory power ($R^{2}=0.019$, p=0.771). Adding perceived spatial environment raised R^2^ to 0.872, with a strongly significant positive coefficient (β=0.933, p<0.001). H2 was therefore supported. Results are reported in Table 7.

### 3.6. Bootstrap Mediation Analyses (Primary)

A parallel-mediation model with five candidate mediators was estimated using non-parametric bootstrap (5000 resamples; bias-corrected 95% Cis), adjusting for age, gender, self-rated health, education, and chronic-disease status. Table 8 presents standardized indirect effects (a × b) with 95% BC Cis.

Two empirical insights warrant explicit emphasis. First, four of the five hypothesized parallel mediators—place attachment, perceived social environment, service environment, and attitudinal preferences—yielded statistically significant indirect effects, with place attachment exhibiting the largest absolute magnitude (a×b=0.257). Second, contrary to H3, the primary behavioral mediator—neighborhood social interaction—failed to attain statistical significance (a×b=0.100; 95% bias-corrected bootstrap CI [−0.000, 0.205], with the lower bound marginally encompassing zero). Consequently, the bootstrapping analysis fails to support H3, while supporting H4 across the four exploratory pathways.

**Figure 3:**
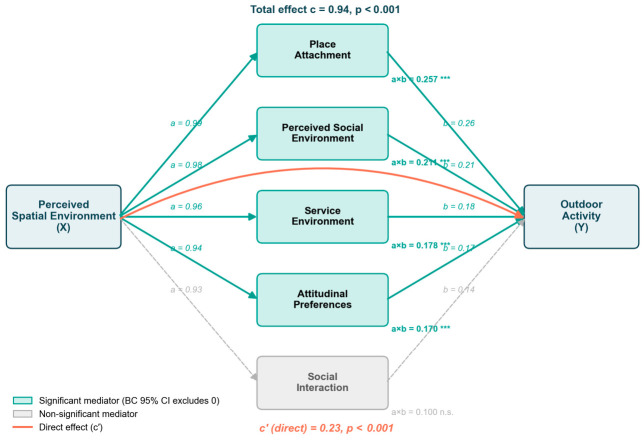
Parallel mediation model linking perceived spatial environment to outdoor activity through five candidate mediators. Standardized coefficients are reported. Indirect effects (a × b) were estimated using 5000-iteration non-parametric bootstrap with bias-corrected 95% confidence intervals. Place attachment, perceived social environment, service environment, and attitudinal preferences emerged as significant mediators (solid green paths), whereas neighborhood social interaction—the a priori hypothesized focal mediator—did not reach significance (dashed grey paths; 95% BC CI [−0.000, 0.205]). Covariates: age, gender, self-rated health, education, and chronic-disease status. *N* = 140. *** indicates BC 95% CI excludes zero; n.s. = not significant.

Consistent with the analytic plan (Section 2.4), these bias-corrected bootstrap estimates—rather than the normal-theory Sobel test—are retained as the basis for inference, because bootstrapping does not assume a normal sampling distribution for the indirect effect and offers better Type I error control in small-to-moderate samples. Full indirect-effect estimates with their bias-corrected confidence intervals are reported in Table A3. Figure 3 visualizes the full parallel-mediation model across all five evaluated pathways.

### 3.7. Mediator Comparison and Theoretical Reframing

Rank-ordering the indirect effects in Table 8 by absolute magnitude uncovers a compelling empirical hierarchy: affective and contextual pathways are larger than the behavioral pathway, whereas the baseline behavioral mediator (social interaction) emerges as the weakest and solitary non-significant path. Specifically, place attachment (a×b=0.257) exerts an indirect effect 2.6 times the magnitude of social interaction (a×b=0.100) which was itself non-significant, maintaining a confidence interval securely bounded away from zero. This ratio should be read descriptively rather than as a precise effect-size comparison. Furthermore, perceived social environment (0.211), service environment (0.178), and attitudinal preferences (0.170) all systematically eclipse social interaction in mediating efficacy.

This empirical configuration necessitates a profound theoretical recalibration. While the initial framework positioned the frequency of enacted social interaction as the foundational behavioral conduit translating environmental perceptions into outdoor engagement, these bootstrapping results favor an affective–cognitive appraisal paradigm. Within this refined schema, place-based affective bonds (place attachment), shared contextual appraisals of the social climate (perceived social environment), and cognitive preference alignment (attitudinal preferences) jointly translate spatial perceptions into behavioral outcomes. This shift aligns robustly with Place Attachment Theory (Scannell & Gifford, 2010; Lewicka, 2011), which prioritizes the meaning–identity–emotion triad over atomized, discrete behavioral counts.

### 3.8. Sensitivity Analysis: Cook’s-D Outlier Removal

To ensure that our substantive conclusions were not artifacts of the 12 high-leverage observations, all primary specifications were re-estimated utilizing the unfiltered Pattern-1 sample (N=152; Table A2). The resulting coefficients and explained variance remained virtually identical to the baseline trimmed models. Specifically, the main regression coefficient shifted negligibly ($\beta_{\mathrm{SPACE}\text{\_}\mathrm{ENV}}=0.956$ for N=152 versus 0.962 for N=140; Δ=0.006), while the model fit exhibited minimal variation (Model $R^{2}=0.925$ for N=152 versus 0.940 for N=140; Δ=0.015). Furthermore, the non-parametric bootstrap mediation analysis replicated the exact configuration of four statistically significant and solitary non-significant pathways across both sample profiles. These results demonstrate that our empirical insights are robust against Cook’s-*D* outlier exclusion. The trimmed sample is consequently retained as the primary analytic dataset, as the targeted exclusion of high-leverage cases optimizes standard error precision without altering any substantive theoretical interpretations.

### 3.9. Discriminant Validity and Common-Method Bias

Evaluating discriminant validity via the Fornell–Larcker criterion (Table A4) revealed that for six of the seven latent constructs, inter-construct correlations (r=0.875–0.968) systematically eclipsed their respective $\sqrt{\mathrm{AVE}}$ values (0.734–0.850). For instance, while perceived spatial environment yielded $\sqrt{\mathrm{AVE}}=0.734$, it covaried substantially with place attachment (r=0.951) and outdoor activity (r=0.968), formally substantiating the conceptual-redundancy concerns articulated. Concurrently, Harman’s single-factor test extracted a primary-factor variance of 48.3%, falling just below Podsakoff et al.’s (2003) 50% critical threshold. Although this precludes catastrophic common-method bias (CMB), it confirms CMB as a non-trivial vector for variance inflation. Ultimately, the confluence of attenuated discriminant validity, persistent CMB, and inflated zero-order correlations suggests that the relative magnitudes of these mediating pathways be interpreted strictly as provisional structural rankings rather than definitive causal estimates. Rigorous multi-method replications—integrating objective GIS spatial data, accelerometer-derived activity metrics, and independent environmental audits—remain needed before drawing policy-actionable conclusions.

Given this pattern, the mediation model is best interpreted as a statistical decomposition of overlapping self-report constructs rather than as evidence of distinct psychological or behavioral pathways. Correlations of this magnitude indicate that the constructs may not be sufficiently distinct, and that shared method variance and item-content overlap may have inflated the observed associations. This does not invalidate the findings, but it does require that the relative magnitudes of the mediating paths be read as provisional structural rankings.

## 4. Discussion

Our analyses are consistent with a robust association between the perceived spatial environment and older adults’ outdoor activity. Even after rigorously partialling out demographic covariates, spatial perception substantially increased the model’s explanatory power and was a strong positive correlate with outdoor engagement. Older adults who appraised their localized spatial conditions more favorably exhibited significantly higher frequencies of outdoor participation. This pattern resonates with the broader environment–behavior literature while introducing a critical behavioral nuance: for older cohorts, outdoor activity is not merely a function of personal motivation or physiological capacity. Rather, it is continuously filtered through a subjective appraisal of whether the surrounding urban fabric is accessible, safe, comfortable, and supportive. Even when objective infrastructural prerequisites are met, perceived inconvenience, safety concerns, or an absence of usable resting nodes can effectively suppress out-of-home behavior. Environmental perception thus operates as a cognitive and behavioral gatekeeper, regulating the translation of physical settings into actualized outdoor excursions. While the cross-sectional, self-reported design necessitates interpretative caution regarding causality, the behavioral implications remain acute: in high-density Chinese metropolises where private space is severely constrained, community-level outdoor areas serve as the primary spatial armature for walking, resting, and informal socializing. Consequently, the perceived quality of these micro-geographies is inextricably tied to their actual utilization by older residents.

The bootstrap analyses did not support the originally hypothesized behavioral pathway (H3). Although the perceived spatial environment was a strong correlate of neighborhood social interaction (H2; Table 7), the indirect effect of spatial perception on outdoor activity through social interaction was not statistically significant once the four affective and contextual mediators and the demographic covariates were modeled simultaneously (a × b = 0.100; 95% BC CI [−0.000, 0.205]). In other words, in this sample the frequency of enacted neighborly contact was not statistically supported as the conduit linking environmental perceptions to out-of-home activity. We therefore describe perceived spatial environment as associated with social interaction, while noting that the indirect pathway through social interaction was not supported. This complicates a straightforward “perception → social contact → activity” reading in dense Chinese communities: a supportive environment may afford abundant casual encounters, yet the ubiquity of such encounters in high-density settings (see the ceiling-effect interpretation below) may limit their incremental contribution. Because the design is cross-sectional and single-source, the strong perceived-environment–interaction association nonetheless leaves open the possibility that social mechanisms operate over longer timeframes, or through qualitative features of contact not captured by a frequency-based measure.

An unanticipated and theoretically relevant finding is the structural divergence between affective-cognitive and behavioral mediators. While place attachment emerged as the dominant mediating pathway (a×b=0.257), the a priori hypothesized behavioral conduit—social-interaction frequency—constituted the sole non-significant vector. This structural inversion of our initial conceptual architecture invites theoretical reframing, interpretable through three distinct mechanisms. First (meaning versus frequency), discretionary outdoor engagement in later life appears proximally governed by profound affective evaluations—emotional bonds and spatial identity—rather than enumerated behavioral counts (Lewicka, 2011; Scannell & Gifford, 2010). Consequently, older adults draw mobility motivation from spatial meaning rather than mere interactive frequency. Second (contextual saturation), the ubiquitous and unavoidable nature of casual encounters in high-density Chinese urban neighborhoods likely induces a ceiling effect, fundamentally compressing variance and attenuating the statistical power necessary to detect differential behavioral impacts. Third (ecological versus enacted networks), the robust mediating efficacy of the perceived social environment indicates that the symbolic climate of the milieu—indexing latent social affordances such as collective efficacy and inclusivity—predicts spatial engagement far better than self-reported enacted frequencies. Collectively, these insights compel a revised theoretical paradigm wherein the perceived environment catalyzes outdoor activity predominantly through affective place bonds and cognitive appraisals of the collective social climate, rather than through the frequency of enacted neighborly interactions. This structural refinement aligns robustly with contemporary place-attachment literature (Lewicka, 2011) and environmental gerontology’s fundamental prioritization of person–environment fit over localized person–person contact (Wahl et al., 2012).

The following implications are framed as suggested directions, contingent on replication of these associations in longitudinal and multi-method studies. They should not be interpreted as evidence that environmental intervention will causally produce the outcomes described. These empirical insights suggest possible directions for design and policy for age-friendly urban renewal. First, morphological enhancements may warrant prioritization. In high-density settings, outdoor activity relies heavily upon the perceived walkability, safety, and comfort of the immediate surroundings. Strategic interventions could prioritize continuous barrier-free pedestrian corridors, slip-resistant surfaces, shaded pathways, adequate illumination, and strategically spaced resting infrastructure. Second, micro-scale public realms warrant disproportionate investment. Pocket parks, building-threshold plazas, and semi-public neighborhood nodes frequently possess outsized behavioral significance for older adults. Their utility is governed less by absolute spatial volume than by proximity, visual permeability, comfort, and usability. In highly densified urban cores where expansive open spaces are ecologically impossible, upgrading these everyday micro-spaces is typically more feasible and behaviorally consequential. Third, physical retrofitting may benefit from being coupled with deliberate social programming. Given that perceived social climate (rather than enacted social interaction) emerged as a significant mediator the environment–activity relationship, morphological upgrades alone are unlikely to fully maximize behavioral shifts. Cultivating morning exercise cohorts, community gardening collectives, and recurring neighborhood events can effectively transfigure upgraded physical spaces into vibrant social settings. Fourth, age-friendly evaluation paradigms must formally integrate subjective perceptual metrics. While prevailing renewal policies often fixate on auditing physical facility inventories, our findings demonstrate that older adults’ subjective spatial appraisals are the proximal drivers of their outdoor behavior. Future assessment protocols must transcend objective checklists to evaluate whether residents genuinely perceive these facilities as accessible, secure, and behaviorally meaningful. These recommendations align seamlessly with the WHO Global Age-Friendly Cities framework (World Health Organization, 2007), China’s Complete Residential Community Construction Guidelines (Ministry of Housing and Urban-Rural Development of the People’s Republic of China, 2021), and broader national initiatives advocating for community-level participation and robust support systems (National Health Commission of the People’s Republic of China & National Working Commission on Aging, 2021).

## 5. Limitations

Several critical methodological parameters constrain the present findings. First, the cross-sectional architecture strictly precludes causal inference, leaving reverse causality—whereby actively mobile older adults systematically cultivate stronger spatial attachments—equally plausible, necessitating future longitudinal or quasi-experimental validation. Second, spatial and demographic selection biases inherently compromise external validity. Because recruitment occurred exclusively at active community nodes within a well-resourced coastal city (Xiamen), the sample systematically excludes homebound populations. Compounded by attrition that skewed the final cohort toward demographically vulnerable profiles (Table A1), the observed associations reflect an already-active urban demographic, precluding seamless generalization to inland, rural, or structurally disparate contexts without rigorous multi-city, residence-based probability sampling. Third, profound measurement artifacts demand acute diagnostic caution. The anomalously elevated inter-construct correlations (r=0.88–0.97) and standardized effects (β≈0.96) signal severely attenuated discriminant validity, as inter-construct covariances systematically eclipsed their respective $\sqrt{\mathrm{AVE}}$ thresholds. Driven by non-trivial common-method bias and inherent semantic redundancies, these artificially inflated $R^{2}$ estimates dictate that mediating hierarchies be interpreted strictly as provisional, hypothesis-generating frameworks until triangulated via objective GIS metrics and accelerometer-derived mobility data. Finally, although the analytic sample (N=140) possessed adequate statistical power for primary medium-effect pathways, acute power deficits for minor associations (required N=273) precluded robust age-stratified moderation analyses, underscoring the imperative for substantially larger cohorts to rigorously disentangle environment–behavior dynamics across distinct late-life strata.

## 6. Conclusions

Leveraging a cross-sectional cohort (N=140) of community-dwelling middle-aged and older adults in Xiamen, China, this study interrogated the nexus between perceived spatial environments and outdoor engagement through a comprehensive parallel-mediation architecture. Three primary insights emerge. First, perceived spatial environments exhibit a robust positive association with outdoor mobility, supporting H1. Second, the a priori hypothesized behavioral conduit—social-interaction frequency—fails to attain statistical significance under rigorous non-parametric bootstrapping, not supporting H3. Third, affective and contextual mechanisms, overwhelmingly dominated by place attachment, constitute the primary mediating pathways, supporting H4 across the exploratory pathways. Theoretically, this pattern suggests that within high-density Chinese urban typologies, affective place bonds and cognitive appraisals of the collective social climate—rather than enacted interactive frequencies—may operate as more proximal correlates linking environmental perceptions into spatial engagement. Practically, these findings advocate an integrative paradigm shift in age-friendly urban renewal, compelling interventions that synthesize morphological design with identity-driven place-making and inclusive social-climate cultivation. Ultimately, severely constrained by cross-sectional limitations, spatial selection biases, attenuated discriminant validity, and singular reliance on self-reporting, these empirical associations must be interpreted strictly as hypothesis-generating frameworks, demanding future longitudinal, multi-method, and multi-city substantiation.

## Figures and Tables

**Table 1:** Demographic characteristics of the sample (*N* = 140).

Variable	Category	*N*	%
**Age**	50–59	38	27.1
	60–69	72	51.4
	70–79	22	15.7
	≥80	8	5.7
**G** **e** **nder**	Male	69	49.3
	Female	71	50.7
**Marital Status**	Married	128	91.4
	Widowed	8	5.7
	Divorced	4	2.9
**Occupation**	Retired employee	67	47.9
	Retired from gov./public institution	16	11.4
	Retired veteran cadre	38	27.1
	No fixed occupation	19	13.6
**Pension/Income**	<3000 yuan	62	44.3
	3000–4999 yuan	52	37.1
	5000–7999 yuan	18	12.9
	≥8000 yuan	8	5.7
**Education**	Primary or below	46	32.9
	Middle school	37	26.4
	Senior high/vocational	28	20.0
	Associate degree	14	10.0
	Bachelor or above	15	10.7
**Living Situation**	Living alone	12	8.6
	With spouse	70	50.0
	With children	36	25.7
	With grandchildren	18	12.9
	Other relatives	4	2.9
**Self-rated health**	Very poor	14	10.0
	Poor	18	12.9
	Average	36	25.7
	Good	56	40.0
	Very good	16	11.4
**Chronic diseases**	No	113	80.7
	Yes	27	19.3

**Table 2:** Descriptive statistics of core variables (*N* = 140).

Variable	Range	Mean	SD	Min	Max	Skewness	Kurtosis
Spatial Environment	1–5	3.550	0.863	1.731	4.346	−1.212	−0.380
Place Attachment	1–5	3.531	0.901	1.333	4.583	−1.124	−0.320
Social Interaction	1–4	2.950	0.860	1.000	4.000	−0.971	−0.582
Outdoor Activity	1–4	2.964	0.842	1.000	3.833	−1.179	−0.323
Attitude and Preference	1–5	3.571	0.865	1.625	4.750	−1.057	−0.400
Social Environment	1–5	3.549	0.924	1.375	4.750	−1.053	−0.397
Service Environment	1–5	3.488	0.899	1.000	4.625	−1.079	−0.240

**Table 3:** Reliability and validity of the measurement scales.

Scale	Items	Cronbach’s α	KMO	Bartlett’s χ^2^	df	*p*
Spatial Environment	26	0.966	0.970	2307.64	325	<0.001
Place Attachment	12	0.931	0.949	917.98	66	<0.001
Social Interaction	5	0.904	0.888	410.50	10	<0.001
Outdoor Activity	6	0.916	0.919	521.60	15	<0.001
Attitude and Preference	8	0.893	0.920	490.34	28	<0.001
Social Environment	8	0.902	0.930	542.19	28	<0.001
Service Environment	8	0.899	0.920	527.84	28	<0.001

**Table 4:** Pearson correlation matrix of core variables (*N* = 140).

Variable	1	2	3	4	5	6	7
1. Spatial Environment	1	—	—	—	—	—	—
2. Place Attachment	0.951 ***	1	—	—	—	—	—
3. Social Interaction	0.928 ***	0.897 ***	1	—	—	—	—
4. Outdoor Activity	0.968 ***	0.946 ***	0.913 ***	1	—	—	—
5. Attitude and Preference	0.937 ***	0.915 ***	0.890 ***	0.929 ***	1	—	—
6. Social Environment	0.931 ***	0.913 ***	0.896 ***	0.935 ***	0.912 ***	1	—
7. Service Environment	0.927 ***	0.910 ***	0.875 ***	0.926 ***	0.899 ***	0.915 ***	1

Note: *** *p* < 0.001.

**Table 5:** Group differences in outdoor activity and social interaction (Note: * *p* < 0.05).

Factor	Dependent Variable	Group Means	Test Statistic	*p*	Levene *p*
Gender	Outdoor Activity	M: 2.96; F: 2.97	*t* = −0.11	0.915	>0.05
Gender	Social Interaction	M: 2.95; F: 2.95	*t* = −0.03	0.977	>0.05
Gender	Spatial Environment	M: 3.52; F: 3.58	*t* = −0.36	0.718	>0.05
Chronic diseases	Outdoor Activity	No: 2.97; Yes: 2.95	*t* = 0.09	0.926	>0.05
Chronic diseases	Social Interaction	No: 2.93; Yes: 3.04	*t* = −0.63	0.527	>0.05
Age	Outdoor Activity	50–59: 2.71; 60–69: 3.12; 70–79: 2.72; ≥80: 3.46	*F* = 3.62	0.015 *	0.000
Age	Social Interaction	50–59: 2.72; 60–69: 3.11; 70–79: 2.65; ≥80: 3.48	*F* = 3.82	0.011 *	0.005
Education	Outdoor Activity	Primary: 3.09; Middle: 3.01; Senior/voc.: 2.74; Assoc.: 2.85; Bachelor+: 2.99	*F* = 0.88	0.481	0.011
Education	Social Interaction	Primary: 3.04; Middle: 3.01; Senior/voc.: 2.66; Assoc.: 2.86; Bachelor+: 3.13	*F* = 1.17	0.326	0.110
Self-rated health	Outdoor Activity	V.poor: 3.10; Poor: 3.10; Avg: 3.01; Good: 2.88; V.good: 2.88	*F* = 0.41	0.800	0.217
Self-rated health	Social Interaction	V.poor: 3.10; Poor: 3.10; Avg: 2.99; Good: 2.86; V.good: 2.89	*F* = 0.44	0.780	0.869
Income	Outdoor Activity	<3000: 2.94; 3000–4999: 2.96; 5000–7999: 2.84; ≥8000: 3.46	*F* = 1.07	0.364	0.006
Income	Social Interaction	<3000: 2.98; 3000–4999: 2.91; 5000–7999: 2.79; ≥8000: 3.35	*F* = 0.85	0.467	0.055

**Table 6:** Hierarchical regression predicting outdoor activity (*N* = 140).

Predictor	Model 1 β	Model 2 β	Model 3 β	Model 4 β
Age	0.123	0.024	0.025	0.027
Gender	0.010	−0.018	−0.015	−0.016
Self-rated health	−0.078	−0.040	−0.036	−0.027
Education	−0.121	−0.041 ^†^	−0.044 *	−0.024
Chronic diseases	−0.072	−0.047 *	−0.051 *	−0.033
Spatial Environment	—	0.960 ***	0.832 ***	0.469 ***
Social Interaction	—	—	0.137 *	0.058
Social Environment	—	—	—	0.118 ^†^
Service Environment	—	—	—	0.096 ^†^
Place Attachment	—	—	—	0.185 **
Attitude and Preference	—	—	—	0.068
R^2^	0.035	0.940	0.942	0.953
Adjusted R^2^	−0.001	0.937	0.939	0.949
ΔR^2^	0.035	0.904	0.002	0.011
*F* (model)	0.98	345.88	307.34	235.99
*F* (change)	0.98	1997.18	5.52	7.37
*p* (change)	0.431	<0.001	0.020	<0.001
Durbin–Watson	1.972	1.973	1.971	2.013

Note: Standardized coefficients are reported. ^†^ *p* < 0.10, * *p* < 0.05, ** *p* < 0.01, *** *p* < 0.001.

**Table 7:** Hierarchical regression predicting social interaction (*N* = 140).

Predictor	Model 1 β	Model 2 β
Age	0.087	−0.010
Gender	0.001	−0.026
Self-rated health	−0.063	−0.026
Education	−0.055	0.022
Chronic diseases	0.008	0.033
Spatial Environment	—	0.933 ***
R^2^	0.019	0.872
Adjusted R^2^	−0.018	0.866
ΔR^2^	0.019	0.853
*F* (model)	0.51	150.83
*F* (change)	0.51	885.73
*p* (change)	0.771	<0.001
Durbin–Watson	1.903	2.028

Note: Standardized coefficients are reported. *** *p* < 0.001.

**Table 8:** Bootstrap parallel-mediation analysis (5000 resamples; bias-corrected 95% Cis).

Mediator	Indirect (a × b)	95% BC CI	Significance
Place Attachment	0.257	[0.131, 0.385]	***
Perceived Social Environment	0.211	[0.107, 0.311]	***
Service Environment	0.178	[0.089, 0.278]	***
Attitudinal Preferences	0.170	[0.052, 0.293]	***
Social Interaction (focal)	0.100	[−0.000, 0.205]	n.s.

Note: *** indicates 95% BC CI excludes zero. *N* = 140.

## Data Availability

The data presented in this study are available upon request from the corresponding author. The data are not publicly available due to privacy and ethical restrictions related to survey participants.

## Appendix A

Perceived Spatial Environment, Mediators, and Outdoor Activity Among Older Adults (*N* = 140).

**Table A1:** Attrition analysis: analytic sample vs. excluded respondents.

Variable	Included (*N* = 140)	Excluded (*N* = 111)	Test Statistic	*p*
Age group	1.99 ± 0.81	1.66 ± 0.76	*t* = 3.30	0.001
Education	2.39 ± 1.31	3.37 ± 1.29	*t* = −5.95	<0.001
Self-rated health	3.31 ± 1.15	3.95 ± 0.84	*t* = −5.08	<0.001
Monthly income	1.79 ± 0.88	2.07 ± 0.90	*t* = −2.47	0.014
Gender	—	—	χ^2^ = 3.11	0.078
Chronic disease	—	—	χ^2^ = 1.22	0.270

Note. Values are M ± SD for ordinal variables (independent-samples *t*-tests); gender and chronic disease are categorical (χ^2^, df = 1). The analytic sample was significantly older, less educated, in poorer self-rated health, and lower income than excluded respondents; gender and chronic-disease distributions did not differ.

**Table A2:** Sensitivity analysis: primary regression with and without Cook’s-*D* exclusion.

Sample	N	β (SPACE_ENV)	*p*	R^2^	*F*
Untrimmed Pattern-1	152	0.956	<0.001	0.925	297.44
Trimmed (primary)	140	0.962	<0.001	0.940	347.82

Note. Standardized OLS coefficient for perceived spatial environment predicting outdoor activity, adjusting for age, gender, self-rated health, education, and chronic-disease status. Excluding the 12 high-leverage cases changed the coefficient negligibly (Δβ = 0.006).

**Table A3:** Bootstrap parallel-mediation indirect effects (5000 resamples; bias-corrected 95% CIs).

Mediator	Indirect (a × b)	95% BC CI	Decision
Place attachment	0.257	[0.131, 0.385]	Supported
Perceived social environment	0.211	[0.107, 0.311]	Supported
Service environment	0.178	[0.089, 0.278]	Supported
Attitudinal preferences	0.170	[0.052, 0.293]	Supported
Social interaction (focal)	0.100	[−0.000, 0.205]	Not supported

Note. *N* = 140; standardized indirect effects; covariates as above. An indirect effect is treated as supported only when the BC 95% CI excludes zero. These values correspond to the primary results in Table 8.

**Table A4:** Bootstrap results of the parallel multiple-mediator model (5000 resamples, bias-corrected 95% CIs).

	1	2	3	4	5	6	7
1. Spatial environment	0.734						
2. Place attachment	0.951	0.756					
3. Social interaction	0.928	0.897	0.850				
4. Outdoor activity	0.968	0.946	0.913	0.841			
5. Attitude & preference	0.937	0.915	0.890	0.929	0.755		
6. Social environment	0.931	0.913	0.896	0.935	0.913	0.771	
7. Service environment	0.927	0.910	0.875	0.926	0.899	0.915	0.766

Note. √AVE values (0.734–0.850) are exceeded by multiple inter-construct correlations (0.875–0.968); the Fornell–Larcker criterion for discriminant validity is therefore not met.
